# No Development of Imipenem Resistance in Pneumonia Caused by *Escherichia coli*

**DOI:** 10.1097/MD.0000000000001020

**Published:** 2015-06-26

**Authors:** Josef Yayan, Beniam Ghebremedhin, Kurt Rasche

**Affiliations:** From the Department of Internal Medicine, Division of Pulmonary, Allergy, and Sleep Medicine, HELIOS Clinic Wuppertal, Witten/Herdecke University, Wuppertal (JY, KR); Institute for Medical Laboratory Diagnostics, Center for Clinical and Translational Research Wuppertal, Witten/Herdecke University, Witten (BG), Germany.

## Abstract

**Background:**

Antibiotic resistance continues to rise due to the increased number of antibiotic prescriptions and is now a major threat to public health. In particular, there is an increase in antibiotic resistance to *Escherichia coli* according to the latest reports.

**Trial Design:**

This article examines, retrospectively, antibiotic resistance in patients with community- and nosocomial-acquired pneumonia caused by *E coli.*

**Methods:**

The data of all patients with community- and nosocomial-acquired pneumonia caused by *E coli* were collected from the hospital charts at the HELIOS Clinic, Witten/Herdecke University, Wuppertal, Germany, within the study period 2004 to 2014. An antibiogram was performed for the study patients with pneumonia caused by *E coli*. Antimicrobial susceptibility testing was performed for the different antibiotics that have been consistently used in the treatment of patients with pneumonia caused by *E coli.* All demographic, clinical, and laboratory data of all of the patients with pneumonia caused by *E coli* were collected from the patients’ records.

**Results:**

During the study period of January 1, 2004 to August 12, 2014, 135 patients were identified with community- and nosocomial-acquired pneumonia affected by *E coli*. These patients had a mean age of 72.5 ± 11.6 (92 [68.1%, 95% CI 60.2%–76.0%] males and 43 [31.9%, 95% CI 24.0%–39.8%] females). *E coli* had a high resistance rate to ampicillin (60.7%), piperacillin (56.3%), ampicillin–sulbactam (44.4%), and co-trimoxazole (25.9%). No patients with pneumonia caused by *E coli* showed resistance to imipenem (*P* < 0.0001).

**Conclusion:**

*E coli* was resistant to many of the typically used antibiotics. No resistance was detected toward imipenem in patients with pneumonia caused by *E coli*.

## INTRODUCTION

*Escherichia coli* is a gram-negative, acid-forming, rod-shaped bacterium with peritrichous flagella that allows it to be motile.^1^*E coli* is the most important representative within the family *Enterobacteriaceae*.^2^*E coli* occurs in the intestinal flora of healthy humans, especially in the colon.^2,3^

The physiological intestinal flora associated with *E coli* strains are facultative pathogenic strains and can cause infections if they enter from the intestine into corresponding regions of the body. *E coli* plays an important role as a frequent cause of bacterial infections, such as urinary tract infections, wound infections, pneumonia, cholecystitis, peritonitis, and gastroenteritis.^4–7^

The detection of intestinal pathogenic *E coli* is difficult. *E coli* is diagnosed by culture of the appropriate isolates and biochemical identification. To detect *E coli*, cell culture assays, enzyme-linked immunosorbent assays, or molecular biological methods are used.^8^ Serotyping remains reserved for specialized laboratories.^9^

Although most *E coli* are harmless, some *E coli* can cause pneumonia.^10^ Pneumonia caused by *E coli* has been neglected with growing frequency over the years.^11^ Meanwhile, *E coli* has become the prominent cause of nosocomial- and hospital-acquired pneumonia in recent years, but very little attention has been given to *E coli* as a cause of community- and hospital-acquired pneumonia.^12^ In the meantime, some strains of *E coli* have developed resistance to commonly used antibacterial drugs.^13^

The prevalence of resistance in *E coli* has deteriorated in recent years in some cases. The proportion of strains with resistance to ampicillin increased in all investigated *E coli* isolates. The most common cause for ampicillin resistance is beta-lactamases, which are largely inhibited by beta-lactamase inhibitors. At the same time, the resistance rate increases in comparison with other antibiotics.^13^ The susceptibility of *E coli* strains to imipenem decreased over recent years.^14^

The early identification of bacteria and determination of their sensitivity to certain antibiotics are important factors in determining the appropriate use of existing antibiotics. For that reason, an investigation was conducted to identify antibiotics that *E coli* was resistant in the last 10 years. Using the hospital database at the HELIOS Clinic, Witten/Herdecke University, in Wuppertal, Germany, data were collected on all of the patients with pneumonia, defined as an acute lower respiratory tract infection caused by *E coli* according to the International Classification of Diseases (ICD) code J15.5.^15,16^

The aim of this study was to investigate antibiotic resistance according to susceptibility testing of the tracheal or bronchial secretions and blood cultures of patients with pneumonia caused by *E coli* over a period of 10 years. Antibiotic use and the failure of antibiotic treatment were monitored in the study population during the study period. The choice of the correct, effective antibiotic against *E coli* should shorten both the duration of patients’ suffering and the length of their hospital stay, as well as reduce patient mortality.

## MATERIAL AND METHODS

### Patients

This quality-control observational study retrospectively examined the resistance to antibiotics in patients with diagnosed community- or nosocomial-acquired pneumonia triggered by *E coli*. Data were collected from hospital charts at the HELIOS Clinic, Witten/Herdecke University, in Wuppertal, Germany, in the study period from January 1, 2004 to September 19, 2014. The study population with community- and nosocomial-acquired pneumonia initiated by *E coli* was mixed in terms of age. All patients over 18 years of age who were detected to have community- or nosocomial-acquired pneumonia caused by *E coli* were included in the study. All of the patients with nosocomial-acquired pneumonia caused by *E coli*, but who were treated initially for other medical reasons in other departments, such as Internal Medicine and Surgery, were included in this study. All of the patients examined at the Department of Neurology who had been suspected of having pneumonia caused by *E coli* were excluded from this study because of restricted access to their patient data.

### Definition of Pneumonia

Pneumonia is an acute inflammation of the lung, primarily affecting the alveoli, which is usually caused by infection from bacteria or viruses and less commonly other microorganisms. Typical clinical symptoms of pneumonia include cough, chest pain, fever, and difficulty in breathing. The diagnosis of pneumonia is performed by X-ray examination and sputum culture.^16,17^

Community-acquired pneumonia caused by *E coli* is an acute infection of the lung parenchyma acquired from normal social contact in the community; this is in contrast to hospital-acquired pneumonia caused by *E coli*, which is acquired during hospitalization.^18^ The classification of pneumonia caused by *E coli* was made in each case, from 2004 to 2014, according to the latest edition of the ICD.^15^

### Tested Antibiotics

The susceptibility to the following antibiotics was tested against *E coli*: ampicillin, piperacillin, ampicillin–sulbactam, piperacillin–tazobactam, cefepime, cefotaxime, ceftazidime, cefuroxime, tetracycline, tigecycline, imipenem, meropenem, ciprofloxacin, levofloxacin, amikacin, gentamicin, tobramycin, co-trimoxazole, colistin, and rifampicin.

The frequency of the use of these antibiotics in clinical practice for the treatment of patients with pneumonia caused by *E coli* was recorded. The frequency of testing of these antibiotics on an antibiogram after detecting microbial *E coli* was noted.

After evaluating the antibiograms of the *E coli* causing pneumonia, the antibiotic that was most commonly used for treatment and most tested for antibiotic susceptibility was compared with the other antimicrobial agents. The antibiotic with the lowest resistance rate was also compared with the other antibiotics tested in the antibiograms.

For *E coli*, inhibition zone diameter breakpoints were used according to the Clinical and Laboratory Standards Institute (CLSI) 2004 to 2011 antibiotic susceptibility testing guidelines.^19^ In 2011, the Europe-wide standards for susceptibility testing (EUCAST) were adopted in place of the CLSI because the EUCAST sets standards for almost all of the pathogens in which our tests are based.^20^ These standards take the clinical and pharmacokinetic aspects of antimicrobial therapy into account more than the previous standards.

### Identification and Antimicrobial Susceptibility Testing

The growth of bacterial isolates was performed on Columbia blood agar and MacConkey agar plates (Becton Dickinson, Heidelberg, Germany) at 37°C for 18 to 48 hours. The identification of the *E coli* isolates by MALDI-TOF MS was performed on a Microflex LT instrument (Bruker Daltonics GmbH, Bremen, Germany) with FlexControl (version 3.0) software (Bruker Daltonics) for the automatic acquisition of mass spectra in the linear positive mode within a range of 2 to 20 kDa, according to the instructions of the manufacturer. The antimicrobial susceptibility testing was performed by use of the automated system BD PHOENIX (Becton Dickinson). In cases of resistance toward carbapenems, the determination of the minimum inhibitory concentration was performed by E-test for these antimicrobials.^21^ The susceptibility results were interpreted according to EUCAST guidelines (breakpoints 2011–2014, www.eucast.org).^20^

### Microbiology

The indication for the performance of a microbiological examination was either routine or explicitly because of a suspected respiratory infection. The secretions from the oral/nasal cavity and trachea were obtained differently depending on the particular case; the commonly used methods applied were bronchoalveolar lavage, tracheal secretions, throat swabs, and sputum collection. The bronchoalveolar lavage was applied in the context of a bronchoscopy. The fiber-optic video bronchoscopies used were OLYMPUS type BF1T180 (Olympus Ltd, Hamburg, Germany) or high-resolution video bronchoscopy PENTAX type EPK-100p (Pentax Europe Ltd, Hamburg, Germany). In each case, about 20 mL of 0.9% saline solution were instilled under local anesthesia and aspirated through the fiber-optic bronchoscope again. The aspirate thus obtained was deposited in 3 different sterile, 40 mL specimen traps (Argyle Specimen Traps, Covidien Germany Ltd, Neustadt/Donau, Germany). Tracheal secretions were also collected by fiber-optic bronchoscopy through aspiration into sterile, 40 mL specimen traps (Argyle Specimen Traps, Covidien Germany Ltd, Neustadt/Donau). The throat swab was collected with a commercial cotton swab transport system (MEUS Srl, Piove di Sacco, Italy) by rotating the swab with slight pressure on the palatal arch of patients with suspected pneumonia. The recovery of sputum was performed by expectoration into a 30 mL sterile sputum collection tube (Salivette, SARSTEDT, Nümbrecht, Germany), which was then sent to the laboratory for analysis.

After the clinical specimens of sputum and tracheal and bronchial secretions were collected, these were transported in suitable containers to the Institute of Medical Microbiology. After propagation of the sputum in a sterile petri dish and testing against a dark background, a macroscopic evaluation was performed to categorize the samples as slimy, purulent, or bloody. Then, a needle was used to separate the bronchial secretions and pus constituents of the saliva. Sputum and tracheal and bronchial secretions were used for microscopic examination, which was conducted after gram staining in 80- to 1000-fold magnification of at least 5 visual fields according to the criteria of Bartlett.^22^ More suspected diagnoses of the pathogen were expressed in the microscopic bacteriological examination than would be expected according to typical morphology and the microbiological infectiological quality standards. Determination was performed of the semiquantitative squamous epithelia, granulocytes, and microorganisms. After that, 3 solid culture media were applied for the cultivation of the most common aerobic, fast-growing microorganisms as a base culture.

Columbia Agar with 5% sheep blood and MacConkey Agar (Becton Dickinson) was incubated at 37°C for 24 to 48 hours as a general culture medium for the growth and discovery of *Streptococcus pneumoniae*, *Streptococcus pyogenes*, *Staphylococcus aureus*, *E coli*, and *Shigella flexneri*. BBL CHROMagar Orientation medium (Becton Dickinson) was used for the detection of *Enterobacteriaceae.* The tested *Enterobacteriaceae* were *E coli*, *Shigella*, *Klebsiella*, *Proteus mirabilis*, *Enterobacter* spp., *Citrobacter* spp., *Serratia marcescens*, *Salmonella*, and *Yersinia*. The medium BBL CDC Anaerobe 5% Sheep Blood Agar (Becton Dickinson) was used for antimicrobial susceptibility testing for the general growth of anaerobes. BD Chocolate Agar (Becton Dickinson) was used as a variant of blood agar for the isolation and cultivation of *Neisseria* and *Haemophilus* species, in which lysis of the erythrocytes was achieved through a brief heating of the agar at 80°C. The lysis caused hemin (factor X) and nicotinamide adenine dinucleotide (factor Y) to be released into the agar and subsequently metabolized by bacteria, resulting in the destruction of the hemolytics as well. BD MacConkey Agar (Becton Dickinson) was used as a selective medium for the detection of gram-negative bacteria. BD Sabouraud Agar (Becton Dickinson) and microscopic analysis were used for the identification of fungi.

### Blood Cultures

Several blood cultures were employed to detect pathogens that propagate through the blood stream. First, skin was carefully disinfected with alcohol (72% ethanol and 10% propan-2-ol) by Bode Cutasept F (Bode Chemie Ltd, Hamburg, Germany). Then, with Braun Injekt single-use syringes (B. Braun Melsungen PLC, Melsungen, Germany), a minimum of 20 mL of blood was taken through venipuncture with a blood-collection needle (Safety-Multifly, SARSTEDT, Nümbrecht, Germany) and injected into 2 specific media—BACTEC Plus Aerobic/F and Plus Anaerobic/F medium (BD, Becton, Dickinson and Company, Heidelberg, Germany) and enriched soybean casein digest broth medium. After injecting the blood culture bottles with new needles, they were sent to the microbiology department where they were entered into a blood culture machine that incubated the specimens at body temperature. The blood culture instrument reported positive blood cultures with bacteria present; most cultures were monitored for 5 days, after which negative vials were removed.

### Laboratory

After the sample collection, the quantitative determination of C-reactive protein in human serum and plasma (the normal value is less than 6 mg/L) was measured in lithium heparin SARSTEDT Monovette 4.7 mL (orange top) using a standard immunoturbidimetric assay on the COBAS 6000 INTEGRA system c 501 (Roche Diagnostics Ltd, Mannheim, Germany). The determination of the leukocyte count (normal range 4000–10,000/μL) in the blood was generally carried out as a routine part of blood counts after collection in EDTA Monovette 2.7 mL by flow cytometry using the Sysmex XE 2100 hematology analyzer (Sysmex Germany Ltd, Norderstedt, Germany).

### Comorbidities

The comorbidities were analyzed in patients with pneumonia caused by *E coli*. Comorbidity was considered the presence of one or more additional disorders existing simultaneously with the primary disease. The additional disorder may also be a behavioral or mental disorder.

Additionally, the length of the hospital stay was assessed in patients with pneumonia caused by *E coli.*

The number of deaths during hospitalization was determined in the study group. The survival analyses were completed using the Kaplan Meier method; the number of days after discharge from the hospital that death occurred was calculated, and the total number of patients in the study group was considered.

### Ethics Statement

The methods of this study were carried out in accordance with the approved institutional guidelines of the Witten/Herdecke University in Germany. All of the patients’ data were anonymized prior to analysis. The Ethics Committee of the Witten/Herdecke University in Germany approved this study and all experimental protocols. Due to the retrospective nature of the study protocol, the Ethics Committee of the Witten/Herdecke University in Germany waived the need for written, informed consent.

### Statistical Analysis

The categorical data were expressed in proportion, while continuous data were expressed as a mean and standard deviation. The calculations were performed at 95% confidence intervals (CIs) for the sex difference of patients with pneumonia caused by *E coli*. A Chi-square test for 2 independent standard normal variables of 3 probabilities was carried out to identify whether *E coli* was sensitive, intermediate, or resistant to antibiotics. A Chi-square analysis was performed using the VassarStats website for statistical computation, created by Richard Lowry of Vassar College in Poughkeepsie, New York, USA.^23^ For the calculation of the *P* value using a 2 × 3 Chi-square test, a contingency table was created containing up to 2 rows and 3 columns. The rows represented the amount of active substance of the antibiotics on antibiograms that was tested against *E coli*; when compared with the other antibiotic substances, ampicillin had the highest resistance rate, while imipenem had low resistance profile. The 3 columns were populated by numbers that categorized the *E coli* as sensitive, intermediary, or resistant to the tested antibiotics, in order to calculate the results. One-way analysis of variance for independent samples was performed to compare the number of samples of each antibiotic that were classified as sensitive, intermediary, or resistant from the antibiograms of the culture media from the patients with pneumonia caused by *E coli*. Two-tailed tests were performed, and a *P* value of <0.05 was considered statistically significant.

## RESULTS

In the hospital database used in this study, 240 (3.5%, 95% CI 3.1%–3.9%) patients were found with pneumonia caused by *E coli* (ICD J15.6). This is compared to 6932 patients in all age groups with pneumonia caused by different types of bacteria who had been treated at the HELIOS Clinic, Witten/Herdecke University, Wuppertal, Germany, during the study period of January 1, 2004 to August 12, 2014.

A total of 135 (1.9%, 95% CI 1.6%–2.2%) of 6932 patients with a mean age of 72.5 ± 11.6 years (92 [68.1%, 95% CI 60.2%–76.0%] males and 43 [31.9%, 95% CI 24.0%–39.8%] females) with pneumonia caused by *E coli* met the inclusion criteria for this trial. The male sex was more likely to suffer from pneumonia caused by *E coli*.

The patients were divided into categorical groups depending on the origin of their pneumonia caused by *E coli*. These groups were community-acquired pneumonia, of which 73 patients belonged (54.1%, 95% CI 45.7%–62.5%); nosocomial-acquired pneumonia, of which 43 patients belonged (31.9%, 95% CI 24.0%–39.8%); and aspiration pneumonia, of which 19 patients belonged (14.1%, 95% CI 8.2%–20.0%).

One hundred five patients were excluded from this study. The reasons for the exclusion of these patients were that they had another infectious disease caused by *E coli* or that access to their patient data at the Department of Neurology was restricted. In addition, patients with pneumonia caused by *E coli* that were under the age of 18 and were treated at the Department of Pediatric and Adolescent Medicine were excluded.

The number of tests for each antibiotic varied in this study because some isolates were examined according to CLSI guidelines, while others, in more recent years, were examined according to EUCAST guidelines. In general, the number of antimicrobial susceptibility tests using CLSI guidelines was higher (Table 1).

**TABLE 1:**
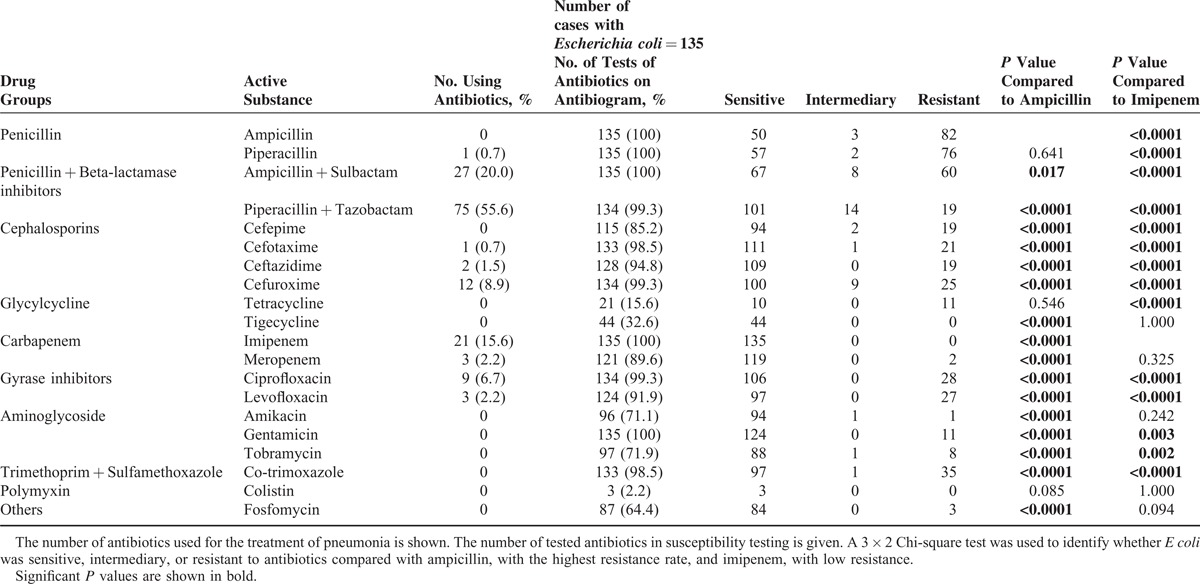
Drug Sensitivity and Drug Resistance in Different Drug Groups in Patients With Pneumonia Caused by *Escherichia coli*

There were highly significant differences with regard to the number of samples classified as sensitive, intermediary, or resistant to a particular antibiotic within the patients with pneumonia caused by *E coli* in this study (*P* < 0.0001). In the susceptibility testing, the mean number of samples tested against antibiotics that were classified as sensitive, intermediary, and resistant was 80.5 ± 39.4, 2.0 ± 3.7, and 21.3 ± 24.2, respectively (Table 1).

The most administered antibiotics in patients with pneumonia caused by *E coli* in this study were the combinations of piperacillin–tazobactam and ampicillin–sulbactam, followed by imipenem (Table 1). No resistance was found to imipenem in any of the patients with pneumonia caused by *E coli* compared with ampicillin; this finding is statistically significant (*P* < 0.0001; Table 1). *E coli* had the highest resistance rate against the antibiotic ampicillin compared with imipenem in this study (*P* < 0.0001; Table 1). *E coli* also had a high resistance rate against piperacillin compared with ampicillin in this investigation (*P* = 0.641; Table 1). The statistical comparison of ampicillin, with the highest rate of resistance, with imipenem, without any rate of resistance, was also determined in this study (*P* < 0.0001, Table 1).

*E coli* was most detected in tracheal secretions, followed by bronchial secretions and sputum (Table 2). The tracheal secretions of patients with pneumonia caused by *E coli* were sent to the Department of Medical Microbiology at the HELIOS Clinic in Wuppertal, Germany, for further investigation into the germs present in the secretions (Table 2). All discovered *E coli* were from isolates of facultative pathogenic *E coli* strains in patients with pneumonia (Table 2).

**TABLE 2:**
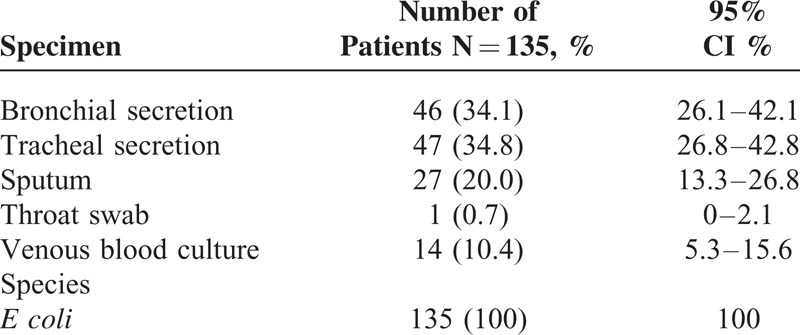
The Various Detection Methods and Species of *Escherichia coli* Bacteria in Patients With Community- and Hospital-Acquired Pneumonia

The amount of C-reactive protein in the serum and plasma of patients with pneumonia caused by *E coli* had a mean value of 89.9 ± 91.7 mg/L. The leukocyte count had a mean value of 12,263 ± 6377.4/μL in the blood of the patients with pneumonia caused by *E coli*.

Most discovered acute comorbidities were cardiac arrhythmias, sepsis, acute respiratory failure, and anemia in patients with pneumonia caused by *E coli* (Table 3). The common chronic comorbidities were hypertension, chronic obstructive pulmonary disease, coronary artery disease, and diabetes in patients with pneumonia caused by *E coli* (Table 3).

**TABLE 3:**
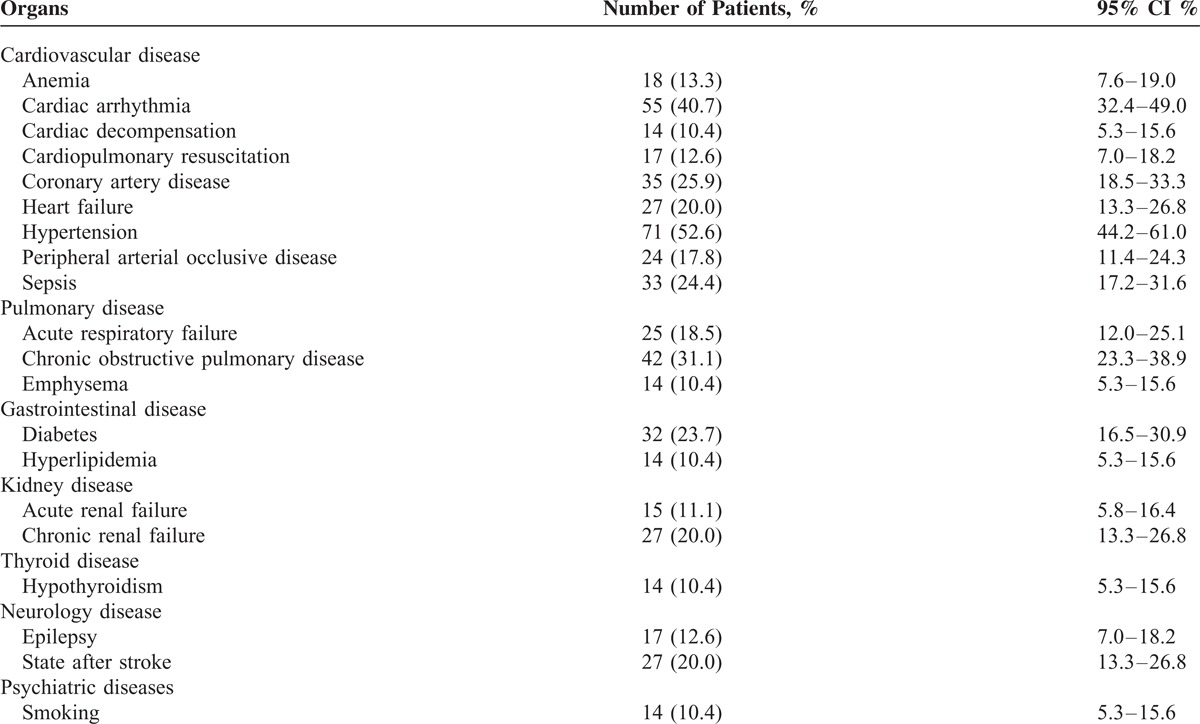
Summary of the Most Common Acute and Chronic Comorbidities in Patients With Pneumonia Caused by *Escherichia coli* Comorbidities Was Not Considered Below 10%

The length of the hospital stay of patients with pneumonia caused by *E coli* had a mean of 18.4 ± 17.3 days.

There were 27 (20.0%, 95% CI 13.3%–26.8%) deaths associated with pneumonia caused by *E coli.* Thus, the survival rate was 80.0% (95% CI 72.5%–87.5%) in patients with pneumonia caused by *E coli* in this study.

## DISCUSSION

During the 10-year study period in this qualitative control observational study, *E coli* developed no resistance to imipenem, an antibiotic used for the treatment of patients with pneumonia. Imipenem is a broad-spectrum antibiotic against aerobic and anaerobic gram-positive and gram-negative pathogens. Imipenem is stable to bacterial beta-lactamases.^24^ In an open prospective study, the efficacy and safety of imipenem has been studied in critically ill patients with nosocomial pneumonia. *E coli* was the most frequently isolated pathogen from tracheobronchial secretions. Imipenem proved to be very effective and relatively well tolerated in the treatment of nosocomial-acquired pneumonia caused by *E coli*.^25^ The clinical efficacy of imipenem for the treatment of aspiration pneumonia caused by *E coli* was also shown in an earlier multicenter study. The efficacy of imipenem monotherapy was found to be very high in cases of aspiration pneumonia caused by *E coli* in this multicenter study.^26^ In another previously conducted multicenter study, the efficacy and safety of imipenem in the treatment of bacterial community- and nosocomial-acquired pneumonia was evaluated. *E coli* was frequently discovered as a cause, among others. The treatment with imipenem was very successful in many of the patients. Resistance to imipenem was detected after the therapy; however, a monotherapy with imipenem was approved as relatively safe and highly effective in severe pneumonia in this previous multicenter study.^27^

Meropenem, another important representative from the antibiotic group of carbapenem, showed very low resistance against *E coli* in this current study. Meropenem is a broad-spectrum antibiotic against aerobic and anaerobic gram-positive and gram-negative bacteria.^28^ Thus, meropenem is still an important option for the empirical treatment of serious community- and nosocomial-acquired pneumonia.^29^

Amikacin had a better resistance rate but lower effectiveness in comparison to meropenem in this present study. It is effective against aerobic gram-negative bacteria.^30^ In a study, the first-line treatment of amikacin was found with a decrease in resistance to other aminoglycosides and a slight increase in the total resistance to amikacin against aerobic gram-negative bacteria.^30^ In general, amikacin is not used as a first-line treatment in the therapy of pneumonia.

A relatively low resistance of *E coli* to fosfomycin was also observed in the *E coli* detected in tracheal secretions of patients with pneumonia in this current study. Fosfomycin is often used because of its good activity against some commonly occurring gram-negative bacteria in nosocomial infections in clinical settings.^31^

Aminoglycoside antibiotics such as tobramycin are also highly effective against gram-negative bacteria according to clinical data in the literature. Previous studies have favored aminoglycosides as monotherapy over beta-lactam monotherapy in gram-negative pneumonia, but there is remarkably little data to suggest the superiority of 2 antibiotics over single agents when they have been compared prospectively.^32^*E coli* was well sensitive and had relative low resistant against tobramycin in this current study.

Another representative of the antibiotic class of aminoglycosides is gentamicin. Gentamicin acts primarily against gram-negative bacteria and also staphylococci. In a previously performed study, antibiotic susceptibility was tested against gram-positive and gram-negative bacteria from patients with purulent infections. Gentamicin was very effective against the main pathogens of purulent infections, including multidrug-resistant *E coli*. Gentamicin was very effective in the treatment of pneumonia, peritonitis, sepsis, postoperative purulent wounds, and urinary tract infections.^33^ In this present study, gentamicin showed a good effectiveness at a relatively low resistance rate in *E coli* in patients with community- and nosocomial-acquired pneumonia.

Tetracycline had the same resistance rate in *E coli* as gentamicin in this study, but the effect of tetracyclines against *E coli* in patients with pneumonia was significantly lower than that of gentamicin. Tetracycline, an antibiotic of the class called glycylcyclines, is still used extensively because of its unusually broad antimicrobial spectrum and its relative safety.^34^ However, tetracycline is not recommended for the empiric treatment of pneumonia caused by *E coli.*^35^

Tigecycline is a first-in-class glycylcyclines with broad spectrum activity. The drug has been used since 2005 for complicated intra-abdominal infections and complicated skin and soft tissue structure infections; it is currently used in the United States for community-acquired pneumonia in adults. Tigecycline has good activity in vitro against a range of gram-positive, gram-negative, and atypical bacteria causing community-acquired pneumonia.^36^ In this current study, *E coli* responded as sensitive to tigecycline in the susceptibility testing of all examined patients with pneumonia, but not all the isolates of tracheal secretions were examined after detecting *E coli* in this study population.

A previous randomized trial compared the safety and efficacy spectrum of cefepime and ceftazidime in the treatment of severe bacterial infections, including sepsis, urinary tract infections, bacterial bronchitis, bacterial pneumonia, and intra-abdominal infections. Most patients had a urinary tract infection, and the most frequently isolated pathogen was *E coli*. The results of the previously conducted study by Huang et al showed that cefepime was an effective antibiotic as ceftazidime in the treatment of severe bacterial infections such as sepsis, urinary tract infections, bacterial bronchitis, bacterial pneumonia, and intra-abdominal infection.^37^

Ceftazidime is a third-generation cephalosporin. The activity of ceftazidime is against gram-negative bacteria, including *P aeruginosa*. Ceftazidime remains an important option for the treatment of hospital-acquired pneumonia.^38^ Cefepime is a broad-spectrum cephalosporin antibiotic of the fourth generation with broad spectrum of activity against gram-positive and gram-negative aerobic bacteria. Cefepime has demonstrated clinical efficacy against a variety of infections, including urinary tract infections, pneumonia, and skin infections.^39^ Cephalosporin antibiotics ceftazidime and cefepime showed a very similar sensitivity and resistance in the drug susceptibility testing against *E coli* from patients with pneumonia in this current study.

The antimicrobial activity of cefuroxime was slightly lower than that of ceftazidime and cefepime in this current study. Cefuroxime is a cephalosporin of the second generation with a broad antimicrobial activity against both gram-positive and gram-negative organisms.^40^ Clinical studies have shown cefuroxime to be effective therapy for infections of the respiratory tract, as well as other infections.^41^

The gyrase inhibitors ciprofloxacin and levofloxacin had the same results in both the spectrum of activity and in the resistance rate against *E coli* in this current study. The resistance rate of ciprofloxacin and levofloxacin was increased in this present study related to previous studies. Ciprofloxacin and levofloxacin are broad-spectrum antibiotics of the fluoroquinolone group against a range of gram-positive and gram-negative bacteria and atypical organisms.^42^

Co-trimoxazole is a combination of trimethoprim and sulfamethoxazole antibiotics. Since the late 1960s, co-trimoxazole was used in clinical practice for the treatment of pneumonia, urinary tract infections, sexually transmitted diseases, gram-negative sepsis, intestinal infections, and typhoid. Several retrospective and prospective studies have demonstrated good clinical results with co-trimoxazole treatment of invasive methicillin-resistant *S aureus* infections.^43^ Co-trimoxazole was effective in over three-quarters of the tested *E coli* detected in tracheal secretions of patients with pneumonia in this current study. *E coli* was resistant to co-trimoxazole in a quarter of the patients with pneumonia in this study. Although the spectrum of activity of co-trimoxazole is against both gram-positive and gram-negative bacteria, co-trimoxazole is not generally used as an empiric treatment of community- and nosocomial-acquired pneumonia in clinical practice.

Among the antibiotic group of penicillin, piperacillin–tazobactam had the lowest resistant rate in *E coli* isolated from tracheal secretions of this study population. Piperacillin–tazobactam had the same effectiveness as some representatives of the cephalosporins, such as cefepime and ceftazidime, in this study. Piperacillin–tazobactam is a commonly prescribed intravenous antibiotic from moderate to severe infections in the hospital, used because of its broad activity against many pathogenic bacteria.^44^

Ampicillin–sulbactam is a beta-lactamase inhibitor combination antibiotic that is commonly used in hospitals against a broad spectrum of aerobic gram-positive and gram-negative and anaerobic bacteria.^45^ Although ampicillin–sulbactam is usually effective against *E coli*, an increase in the rate of resistance has been described in previous studies.^46,47^ A high variability in resistance frequencies to the beta-lactam inhibitor combination ampicillin–sulbactam was also observed in this study. Resistance to beta-lactamase inhibitor combinations in *E coli* isolates has been widely reported.^48,49^

Piperacillin is a new generation of semisynthetic penicillins. It has a broad spectrum of activity against gram-positive and gram-negative aerobic and anaerobic bacteria. Although piperacillin has shown higher activity against beta-lactamase producing organisms than the other penicillins;^50^ in this study, piperacillin was less effective toward *E coli* from the tracheal secretions of patients with pneumonia. Piperacillin showed a high resistance rate in *E coli* in the susceptibility testing in this study.

Ampicillin has effectiveness against gram-positive organisms as well as some gram-negative bacteria; therefore, ampicillin is referred to as a broad-spectrum antibiotic.^51^ However, ampicillin had the highest rate of resistance in *E coli* in patients with pneumonia in this current study. The *E coli* from the tracheal secretions of patients with pneumonia in this study was less sensitive to ampicillin in the susceptibility testing.

The prevalence of antibiotic resistance in *E coli* is increasing rapidly. Excessive use of antibiotics may promote the emergence and spread of resistant microorganisms. Consistent infection control measures and modification of antibiotic use patterns limit or reduce the prevalence of resistant organisms.

### Study Limitations

This study describes the situation of *E coli* resistance in a single hospital, so the results cannot be generalized to other geographic locations. After an evaluation of this study, it became apparent that not all antibiotics were tested with the same frequency in the antibiograms of patients with pneumonia caused by *E coli*. The author was unable to clarify whether or not all of these antibiotics were tested for each *E coli* isolate.

## CONCLUSIONS

All of the patients with pneumonia caused by *E coli* showed resistance to a variety of antibiotics. No patients with pneumonia caused by *E coli* showed resistance to imipenem. A similarly good result was found for tigecycline in the susceptibility testing toward *E coli*, but not all patients were tested against tigecycline. All common antibiotics, such as those in this study, should be tested for susceptibility in the case of identification of *E coli* on culture media from all patients with pneumonia, both for the actual drug treatment of patients with pneumonia caused by *E coli* and for monitoring the trend in antibiotic resistance in *E coli* in the future.

## References

[1] Croxen MA Law RJ Scholz R Recent advances in understanding enteric pathogenic *Escherichia coli*. *Clin Microbiol Rev* 2013; 26:822–880. 24092857 10.1128/CMR.00022-13 PMC3811233

[2] Allocati N Masulli M Alexeyev MF Escherichia coli in Europe: an overview. *Int J Environ Res Public Health* 2013; 10:6235–6254. 24287850 10.3390/ijerph10126235 PMC3881111

[3] Kaper JB Nataro JP Mobley HL Pathogenic *Escherichia coli*. *Nat Rev Microbiol* 2004; 2:123–140. 15040260 10.1038/nrmicro818

[4] Steenbergen SM Vimr ER Chromatographic analysis of the Escherichia coli polysialic acid capsule. *Methods Mol Biol* 2013; 966:109–120. 23299731 10.1007/978-1-62703-245-2_7 PMC4085740

[5] Köhler CD Dobrindt U What defines extraintestinal pathogenic *Escherichia coli*? *Int J Med Microbiol* 2011; 301:642–647. 21982038 10.1016/j.ijmm.2011.09.006

[6] Johnson JR Menard M Johnston B Epidemic clonal groups of *Escherichia coli* as a cause of antimicrobial-resistant urinary tract infections in Canada, 2002 to 2004. *Antimicrob Agents Chemother* 2009; 53:2733–2739. 19398649 10.1128/AAC.00297-09 PMC2704706

[7] Rodríguez-Angeles G [Principal characteristics and diagnosis of the pathogenic groups of *Escherichia coli*]. *Salud Publica Mex* 2002; 44:464–475.[Article in Spanish]. 12389490

[8] Vila J Alvarez-Martínez MJ Buesa J [Microbiological diagnosis of gastrointestinal infections]. *Enferm Infecc Microbiol Clin* 2009; 27:406–411.[Article in Spanish]. 19477556 10.1016/j.eimc.2008.11.009 PMC7103285

[9] Boxrud D Monson T Stiles T The role, challenges, and support of pulsenet laboratories in detecting foodborne disease outbreaks. *Public Health Rep* 2010; 125 (Suppl 2):57–62. 20518445 10.1177/00333549101250S207 PMC2846803

[10] Menon RU George AP Menon UK Etiology and anti-microbial sensitivity of organisms causing community acquired pneumonia: a single hospital study. *J Family Med Prim Care* 2013; 2:244–249. 24479091 10.4103/2249-4863.120728 PMC3902680

[11] Ruiz LA Gómez A Jaca C Bacteraemic community-acquired pneumonia due to Gram-negative bacteria: incidence, clinical presentation and factors associated with severity during hospital stay. *Infection* 2010; 38:453–458. 20878457 10.1007/s15010-010-0058-4

[12] Ruiz LA Zalacain R Gómez A *Escherichia coli*: an unknown and infrequent cause of community acquired pneumonia. *Scand J Infect Dis* 2008; 40:424–427. 18418804 10.1080/00365540701732913

[13] Pitout JD Extraintestinal pathogenic *Escherichia coli*: an update on antimicrobial resistance, laboratory diagnosis and treatment. *Expert Rev Anti Infect Ther* 2012; 10:1165–1176. 23199402 10.1586/eri.12.110

[14] Hamouche E Sarkis DK [Evolution of susceptibility to antibiotics of Escherichia coli, Klebsiella pneumoniae, Pseudomonas aeruginosa and Acinetobacter baumanii, in a University Hospital Center of Beirut between 2005 and 2009.]. *Pathol Biol (Paris)* 2012; 60:e15–e20.[Article in French.]. 21719212 10.1016/j.patbio.2011.03.011

[15] World Health Organization (WHO). International Classification of Diseases (ICD). http://www.who.int/classification/icd/en/ Accessed March 9, 2015.

[16] Niederman MS Mandell LA Anzeuto A Guidelines for the management of adults with community-acquired pneumonia. Diagnosis, assessment of severity, antimicrobial therapy, and prevention. *Am J Respir Crit Care Med* 2001; 163:1730–1754. 11401897 10.1164/ajrccm.163.7.at1010

[17] Sahm DF Brown NP Thornsberry C Antimicrobial susceptibility profiles among common respiratory tract pathogens: a GLOBAL perspective. *Postgrad Med* 2008; 120 (3 Suppl 1):16–24. 18931467 10.3810/pgm.2008.09.suppl52.280

[18] Watkins RR Lemonovich TL Diagnosis and management of community-acquired pneumonia in adults. *Am Fam Physician* 2011; 83:1299–1306. 21661712

[19] CLSI, Clinical and Laboratory Standards Insitute. Performance Standards for Antimicrobial Susceptebility Testing: Twenty-second Informational Supplement M100-S22. CLSI, Wayne, PA, USA; 2012.

[20] European Committee on Antimicrobial Susceptibility Testing (EUCAST) breakpoints 2011–2014. http://www.eucast.org Accessed March 9, 2015.

[21] Bauer AW Kirby WM Sherris JC Antibiotic susceptibility testing by a standardized single disk method. *Am J Clin Pathol* 1966; 45:493–496. 5325707

[22] Bartlett JG Diagnosis of bacterial infections of the lung. *Clin Chest Med* 1987; 8:119–134. 3552385

[23] VassarStats [website for statistical computation.] and Concepts & Applications of Inferential Statistics [companion textbook.]. http://vassarstats.net/and http://vassarstats.net/textbook/ Accessed March 9, 2015.

[24] Amin NM New antibiotics: carbapenems, monobactams and quinolones. *Am Fam Physician* 1988; 38:125–134. 3051970

[25] Unertl K Lenhart FP Ruckdeschel G [Treatment of bronchopulmonary infections in patients during artificial respiration with imipenem/cilastatin.]. *Immun Infekt* 1986; 14:229–231.[Article in German.]. 3100428

[26] Kikuchi N Onozaki I Kohno N [Clinical evaluation of therapy for aspiration pneumonia with imipenem/cilastatin sodium.]. *Jpn J Antibiot* 1990; 43:23–30.[Article in Japanese.]. 2112207

[27] Salata RA Gebhart RL Palmer DL Pneumonia treated with imipenem/cilastatin. *Am J Med* 1985; 78:104–109. 3859208 10.1016/0002-9343(85)90110-x

[28] Goncalves-Pereira J Silva NE Mateus A Assessment of pharmacokinetic changes of meropenem during therapy in septic critically ill patients. *BMC Pharmacol Toxicol* 2014; 15:21. 24731745 10.1186/2050-6511-15-21 PMC4006523

[29] Baldwin CM Lyseng-Williamson KA Keam SJ Meropenem: a review of its use in the treatment of serious bacterial infections. *Drugs* 2008; 68:803–838. 18416587 10.2165/00003495-200868060-00006

[30] Ristuccia AM Cunha BA An overview of amikacin. *Ther Drug Monit* 1985; 7:12–25. 3887667 10.1097/00007691-198503000-00003

[31] Michalopoulos AS Livaditis IG Gougoutas V The revival of fosfomycin. *Int J Infect Dis* 2011; 15:e732–e739. 21945848 10.1016/j.ijid.2011.07.007

[32] Saavedra S Vera D Ramírez-Ronda CH Susceptibility of aerobic gram-negative bacilli to aminoglycosides. Effects of 45 months of amikacin as first-line aminoglycoside therapy. *Am J Med* 1986; 80:65–70. 3014877 10.1016/0002-9343(86)90481-x

[33] Bogomolova NS Rodoman VE Kozlov VA [Effectiveness of gentamicin sulfate in suppurative-inflammatory processes of varying localization.]. *Antibiotiki* 1977; 22:558–564.[Article in Russian.]. 329751

[34] Cunha BA Comer JB Jonas M The tetracyclines. *Med Clin North Am* 1982; 66:293–302. 7038336 10.1016/s0025-7125(16)31461-4

[35] Oncu S Erdem H Pahsa A Therapeutic options for pneumococcal pneumonia in Turkey. *Clin Ther* 2005; 27:674–683. 16117975 10.1016/j.clinthera.2005.06.009

[36] Jones CH Petersen PJ Tigecycline: a review of preclinical and clinical studies of the first-in-class glycylcycline antibiotic. *Drugs Today (Barc)* 2005; 41:637–659. 16389407 10.1358/dot.2005.41.10.937460

[37] Huang CK Chen YS Lee SS Safety and efficacy of cefepime versus ceftazidime in the treatment of severe infections. *J Microbiol Immunol Infect* 2002; 35:159–167. 12380788

[38] Gentry LO Antimicrobial activity, pharmacokinetics, therapeutic indications and adverse reactions of ceftazidime. *Pharmacotherapy* 1985; 5:254–267. 3906585 10.1002/j.1875-9114.1985.tb03424.x

[39] Wynd MA Paladino JA Cefepime: a fourth-generation parenteral cephalosporin. *Ann Pharmacother* 1996; 30:1414–1424. 8968455 10.1177/106002809603001211

[40] Smith BR LeFrock JL Cefuroxime: antimicrobial activity, pharmacology, and clinical efficacy. *Ther Drug Monit* 1983; 5:149–160. 6349019

[41] Gold B Rodriguez WJ Cefuroxime: mechanisms of action, antimicrobial activity, pharmacokinetics, clinical applications, adverse reactions and therapeutic indications. *Pharmacotherapy* 1983; 3:82–100. 6344037

[42] Labreche MJ Frei CR Declining susceptibilities of gram-negative bacteria to the fluoroquinolones: effects on pharmacokinetics, pharmacodynamics, and clinical outcomes. *Am J Health Syst Pharm* 2012; 69:1863–1870. 23111670 10.2146/ajhp110464

[43] Goldberg E Bishara J Contemporary unconventional clinical use of co-trimoxazole. *Clin Microbiol Infect* 2012; 18:8–17. 21851485 10.1111/j.1469-0691.2011.03613.x

[44] Hayashi Y Roberts JA Paterson DL Pharmacokinetic evaluation of piperacillin-tazobactam. *Expert Opin Drug Metab Toxicol* 2010; 6:1017–1031. 20636224 10.1517/17425255.2010.506187

[45] Shungu DL Ponticas S Gill CJ Comparative activity of cefoxitin, ampicillin/sulbactam, and imipenem against clinical isolates of *Escherichia coli* and *Klebsiella pneumoniae*. *Clin Ther* 1989; 11:315–318. 2663161

[46] Kaye KS Harris AD Gold H Risk factors for recovery of ampicillin-sulbactam-resistant *Escherichia coli* in hospitalized patients. *Antimicrob Agents Chemother* 2000; 44:1004–1009. 10722504 10.1128/aac.44.4.1004-1009.2000 PMC89805

[47] Oliver A Pérez-Vázquez M Martínez-Ferrer M Ampicillin-sulbactam and amoxicillin-clavulanate susceptibility testing of *Escherichia coli* isolates with different beta-lactam resistance phenotypes. *Antimicrob Agents Chemother* 1999; 43:862–867. 10103192 10.1128/aac.43.4.862 PMC89218

[48] Mendonça N Leitão J Manageiro V Spread of extended-spectrum beta-lactamase CTX-M-producing *Escherichia coli* clinical isolates in community and nosocomial environments in Portugal. *Antimicrob Agents Chemother* 2007; 51:1946–1955. 17371815 10.1128/AAC.01412-06 PMC1891395

[49] Thomson KS Weber DA Sanders CC Beta-lactamase production in members of the family Enterobacteriaceae and resistance to beta-lactam-enzyme inhibitor combinations. *Antimicrob Agents Chemother* 1990; 34:622–627. 2344169 10.1128/aac.34.4.622 PMC171654

[50] 1984; Holmes B Richards DM Brogden RN Piperacillin A review of its antibacterial activity, pharmacokinetic properties and therapeutic use Drugs. 28:375–425. 10.2165/00003495-198428050-00002 6391888

[51] Neu HC Aminopenicillins – clinical pharmacology and use in disease states. *Int J Clin Pharmacol Biopharm* 1975; 11:132–144. 1095502

